# CareHPV, Papanicolaou Positivity Status, and Their Association With Behavioral Risk Factors in Rural Women of Kamrup District, Assam, India

**DOI:** 10.1002/cnr2.70097

**Published:** 2024-12-26

**Authors:** Pallavi Sarma, Debabrata Barmon, Avdhesh Kumar Rai, Amal Chandra Kataki, Anupam Sarma, Upasana Baruah, Lopamudra Kakoti, Debanjana Barman, Ratnadeep Sharma

**Affiliations:** ^1^ DBT Centre for Molecular Biology and Cancer Research Dr B. Borooah Cancer Institute Guwahati Assam India; ^2^ Department of Gynaecologic Oncology Dr B. Borooah Cancer Institute Guwahati Assam India; ^3^ Dr B. Borooah Cancer Institute Guwahati Assam India; ^4^ Department of Oncopathology Dr B. Borooah Cancer Institute Guwahati Assam India; ^5^ Department of Cancer Epidemiology and Biostatistics Dr B. Borooah Cancer Institute Guwahati Assam India; ^6^ Population Based Cancer Registry Dr B. Borooah Cancer Institute Guwahati Assam India

**Keywords:** Assam, careHPV, high‐risk HPV, PAP, rural women

## Abstract

**Background and Objectives:**

Screening of rural women of Assam by careHPV test for high‐risk HPV (hr‐HPV) DNA and Papanicolaou (PAP) test for abnormal cytology.

**Method:**

This prospective cross‐sectional study included 480 non‐pregnant women participants aged 20–70 years from Kamrup District, Assam. Two cervical scrap samples were obtained from eligible enrolled women. The Hr‐HPV DNA test by CareHPV was performed with one cervical scrap, and a second cervical scrap sample was used for the Papanicolaou (PAP) test. The statistical analysis was done using RStudio for variables. A *p*‐value < 0.05 was considered to be statistically significant.

**Results:**

Women having positive hr‐HPV DNA outcomes were 3.33% (16/480) and 7.7% (37/480) women had positive PAP. Tobacco chewing was significantly associated with positive hr‐HPV DNA (*p* = 0.04) and positive PAP (*p* = 0.03) status. Alcohol‐consuming women have a significantly higher risk of positive hr‐HPV DNA (*p* < 0.00001) and positive PAP (*p*‐0.04) outcomes. Irregular menstruation (*p* = 0.004) and urogenital tract infection (*p* = 0.008) also have significant risk for a positive hr‐HPV DNA status. The positive hr‐HPV DNA status was also significant in women having > 3 numbers of children birth (*p* = 0.003).

**Conclusion:**

We found that the positive hr‐HPV DNA status among rural women in Kamrup, Assam, was significantly associated with alcohol consumption, tobacco chewing, irregular menstruation, urogenital tract infection, and more than three children birth. The abnormal cytology outcome was also substantially associated with tobacco chewing and alcohol consumption.

## 1 Introduction

Cervical cancer poses a substantial health burden for women worldwide, with a specific focus on India. In 2020, the global incidence of cervical cancer was 604 127 cases, with a corresponding mortality rate of 341 831. In that year, India had a substantial burden, representing 20% (123 907) of the new cases and 22% (77 348) of the fatalities attributed to cervical cancer [1]. The age‐adjusted incidence rate (AAR) of cervical cancer in North‐East India varies from 4.8 to 27.7 per 100 000 people. The Papumpare district has the highest AAR (27.7), followed by Aizawl district (27.4), Mizoram state (23.2), and Pasighat (20.3) [2]. Among North‐East Indian females in Mizoram state, the probable risk of developing any form of cancer over a lifetime (0–74 years) is the highest (1 in 5 females), followed by Kamrup urban district (1 in 6 females) and Meghalaya (1 in 9 females) [3].

Successful utilization of the Papanicolaou test (PAP) enables the early identification, diagnosis, and appropriate treatment of cervical intraepithelial neoplasia (CIN) lesions, effectively preventing cervical cancer. Many highly industrialized countries have implemented the PAP cytology test as a main screening procedure due to its cost‐effectiveness and simplicity. As a result, the incidence of invasive cervical cancer has decreased by 50%–70%, and the death rate from cervical cancer has decreased by up to 80% [4].

In limited/poor‐resource countries, screening initiatives for cytology‐based tests face several problems. These include complicated procedures that need to be followed (like multiple visits and frequent testing), the need for skilled technicians and pathologists, and the fact that the test is only moderately sensitive even when conditions are perfect [5]. Invasive cervical cancer has been consistently linked to persistent high‐risk human papillomavirus (hr‐HPV) infection, which is a well‐established risk factor [6]. Performing a sufficiently sensitive hr‐HPV DNA test one or two times in a woman's lifespan, as opposed to repeated screening with a moderately sensitive cytology‐based test, may be a more successful strategy for preventing cervical cancer in limited/poor‐resource countries [7].

An Indian microsimulation model study from 2017 to 2026 analyzed the effects of several implementation scenarios on HPV infection and cervical cancer. The research indicated that promptly adopting a two‐visit technique would yield substantial advantages. By incorporating HPV testing immediately every five years during a single appointment, it is possible to prevent 574 100 new instances of cervical cancer and 382 500 deaths among a population of 814 lakh women aged 30 to 34. Nevertheless, deferring the implementation for five years with only one visit will have distinct consequences, even in such a scenario. The HPV test has the potential to prevent 209 300 new cases of cervical cancer and 139 100 deaths [8].

Hybrid Capture 2 (HC2) is a clinically validated screening tool that uses signal amplification technology to detect DNA from 13 high‐risk strains of HPV (16, 18, 31, 33, 35, 39, 45, 51, 52, 56, 58, 59, and 68). This technique has been widely employed in numerous studies worldwide, including in India. Studies conducted in different parts of India have demonstrated hr‐HPV‐positive rates ranging from 17.1% to 6% [9, 10, 11, 12, 13, 14, 15]. The CareHPV Test is based on the same principle as the HC2 Test but due to its simplicity, it can be performed by less skilled technicians in rural areas and doesn't require typical laboratory infrastructure like electricity, water, etc. Indian studies using careHPV tests reported hrHPV positivity from 2.9% to 8.6% [16, 17, 18, 19, 20].

Most cervical cancer diseases among rural women are diagnosed at an advanced stage in India. Insufficient data are available from North‐East India about the frequency of hr‐HPV and CIN lesions and their relationship with risk factors for cervical cancer. We conducted this study on asymptomatic women residing in rural areas of the Kamrup district in Assam, India. The objective was to ascertain the frequency of hr‐HPV DNA by employing the CareHPV test and to evaluate the existence of CINs by utilizing the PAP test. Furthermore, the study sought to investigate the correlation between these findings and different risk variables.

## 2 Materials and Method

We enrolled study participants with informed written consent from cervical cancer awareness and screening camps held in rural regions of the Kamrup district, Assam, India, between April 2017 and December 2020. We included 480 (*n* = 480) eligible participants, non‐pregnant married women between the ages of 20 and 70 out of 500 women who participated in the awareness and screening camps. Cervical samples were collected by medic from all the participants. We excluded (*n* = 20) women with CIN history, exposure to pelvic radiation, hysterectomy, or any other co‐morbid condition, those unable to provide informed permission, and those lacking demographic or behavioural information. The proforma questionnaire included personal interviews to collect information on socio‐demographic characteristics, sexual practices, reproductive health, and tobacco and alcohol habits. This was a community‐based cross sectional study, and a convenient sampling approach was followed. This study was approved by the Institutional Ethics Committee.

### 2.1 CareHPV Test

A CareBrush (Qiagen, USA) measuring 1–1.5 cm was gently inserted into the cervix. The brush was then rotated three clockwise spins to collect the cervical scrap. The scrap was collected in the careHPV (Qiagen, USA) collecting medium, which measured 1 mL. The samples were properly labelled, transported from camp to the lab in a storage box at 15°C–25°C, and stored at 2°C–8°C for 30 days or at −20°C for a longer time. A total of 480 samples were processed for careHPV testing.

Reagents were prepared and used as per the manufacturer's instructions. Briefly, 50 μL of sample from the careHPV collection medium was transferred to the microplate containing 25 μL of reagent1 and complete testing procedure for the careHPV test kit (Qiagen, USA) was done by the careHPV system (Qiagen, USA) according to the manufacturer's instructions. CareHPV utilizes in vitro nucleic acid hybridization to qualitatively detect 14 high‐risk HPV genotypes (16, 18, 31, 33, 35, 39, 45, 51, 52, 56, 58, 59, 66, 68) through signal amplification. The DNA in the specimens was specifically hybridized to an RNA probe for the HPV. The resulting RNA–DNA hybrids were then captured and identified using a microplate chemiluminescence technique using a luminometer. CareHPV assesses the proportion of the relative light unit (RLU) to a positive cut‐off (CO) value (corresponding to 1.0 pg/mL or 5000 viral copies). It provides a binary outcome of either positive or negative, without indicating the specific intensity of the luminescent signal measured in the RLU. Samples were displayed in yellow plus (+) symbol as positive for hr‐HPV when the ratio of RLU/CO was ≥ 1 and the green colour display as negative when ratio of RLU/CO was < 1. The high‐risk HPV calibrator consisted of 1 picogram per millilitre of cloned HPV 16 and carrier DNA. The negative calibrator consisted solely of carrier DNA.

### 2.2 PAP Test

Cervical scrap was obtained by brushing both the outer and inner parts of the cervix and transferring it onto a glass slide. The slide was then treated with ethanol to preserve the cells for cytological examination using the PAP test. The Bethesda system of cytology reporting was employed, which includes the following categories: NILM (negative for intraepithelial lesions and malignancy), encompassing inflammatory and atrophic changes; ASCUS (atypical squamous cells of undetermined significance); LSIL (low‐grade squamous intraepithelial lesions); and HSIL (high‐grade squamous intraepithelial lesions). Women positive for ASCUS, LSIL, and HSIL on their PAP test were categorized as positive. Women positive for PAP (ASCUS, LSIL, HSIL) were advised for colposcopy examination and colposcopy‐guided biopsy, if necessary.

### 2.3 Hybrid Capture2 (HC2) Test

All CareHPV tested samples (positive and equal number of negative samples) have been retested by the HC2 test for semi‐quantitative RLU values for each tested sample. Samples with positivity in both (CareHPV and HC2) tests were scored as positive. The HC2 assay is based on the same principle as careHPV and used for qualitative detection of 13 types of hr‐HPV (16, 18, 31, 33, 35, 39, 45, 51, 52, 56, 58, 59, 68) by signal amplification in microplate chemiluminescence by Luminometer (DML2000). Briefly, 500 μL of specimens from the careHPV collection medium (Qiagen, USA) were transferred into a fresh tube containing 250 μL denaturing solution and the testing protocol for digene HC2 High‐Risk HPV DNA test (Qiagen, USA) was followed as per manufacturer's instructions. The light signals were quantified as RLUs, with the intensity values of the light indicating whether the target DNA was present or not in the tested samples. RLU values that are equal to or more than the CO value (RLU/CO ≥ 1) indicate the presence of hrHPV DNA in the sample. The result of the RLU/CO < 1 test indicated that there was no presence of particular hrHPV DNA or that the level of hrHPV DNA was below the detectable threshold. If the RLUs are equal to the CO, it indicates the presence of about 5000 viral copies in the specimen. The quality control for high‐risk HPV consisted of 5 pg/mL of cloned HPV 16 and carrier DNA. The high‐risk HPV calibrator included 1 picogram per millilitre of cloned HPV 16 and carrier DNA. The carrier DNA served as the negative calibrator. Additionally, two control samples were included, consisting of proven cases of cervical cancer with hr HPV positivity. The test results were generated according to the format of the Hybrid Capture software version 2.0.

### 2.4 Statistical Analysis

In this study, we used statistical analysis to examine the associations between various factors and hr‐HPV as well as PAP test outcomes. All analyses was conducted using RStudio (Version 1.2.1335). We employed several R packages, including pROC, stats, car, MASS and psych. For bivariate analysis, Fisher's exact test was applied to evaluate the relationships between individual variables. To assess multiple variables simultaneously, we performed multivariable binary logistic regression using a backward stepwise approach. This method allowed for the sequential removal of non‐significant variables, refining the model for more precision. Statistical significance was set at *p* < 0.05.

## 3 Results

We conducted a study involving 500 women, but we omitted 20 of them from the analysis due to missing data (*n* = 20). The missing data included factors such as informed consent, an incomplete proforma questionnaire for habit, reproductive health, etc. Hence, a total of 480 women who met the eligibility requirements had their cervical scrap samples processed and analyzed for careHPV and PAP tests. All the female participants were married. The study's participants ranged in age from 20 to 70 years old. The distribution of ages was as follows: 0.6% (*n* = 3) were 20 years old, 16.8% (*n* = 81) were between 21 and 30 years old, 34.3% (*n* = 164) were between 31 and 40 years old, 33.5% (*n* = 161) were between 41 and 50 years old, 11.8% (*n* = 58) were between 51 and 60 years old, and 2.7% (*n* = 13) were between 61 and 70 years old. The educational profile is as follows: 10.2% of individuals are graduates, 21.2% have completed higher secondary education, 38.3% have completed primary education, and 30.2% have not specified their educational level. The prevalence of tobacco chewers was 63.7%. 22.0% of individuals consumed an alcoholic beverage. 31.6% of individuals use contraceptives; 88.8% of individuals reported experiencing regular menstruation, and 33.7% of cases had a history of urinary tract infection. In 53.5% of cases, the age at which individuals got married was below 20 years, while in 42.2% of cases, it fell between the range of 21 and 30 years; 39.0% of individuals experienced their first pregnancy at an age younger than 20 years, while 48.5% had their first pregnancy between the ages of 21 and 30 years. Out of all the participants, 5% did not have any children, while 61.7% had 1–2 children and 33.3% had 3 or more children (Table 1).

**TABLE 1:** Sociodemographic, behavioral characteristics, and risk factor association with hr‐HPV infection (bivariate analysis).

Characteristics	HPV negative	HPV positive	*p* ^a^
Age			
20–30	43	1	0.24
30–40	158	5	
40–50	162	3	
50–60	77	6	
60–70	24	1	
Tobacco habit			
No	302	4	0.002^b^
Yes	162	12	
Betel nut habit			
No	154	4	0.59
Yes	310	12	
Alcoholic beverages			
No	372	2	< 0.00001^b^
Yes	92	14	
Use of contraceptives			
No	316	12	0.78
Yes	148	4	
Urogenital tract infection			
No	312	6	1.00
Yes	152	10	
Urogenital tract infection of husband			
No	453	15	0.33
Yes	11	1	
Regularity of the menstrual cycle			
Irregular	48	6	0.005^b^
Regular	416	10	
Use of sanitary pad			
Yes	288	9	0.61
No	176	7	
Age at the first pregnancy			
10–20	146	7	0.64
20–30	249	7	
30–40	48	1	
40–50	1	0	
NA	0	0	
Total number of children			
< 3	294	3	0.001^b^
≥ 3	148	12	
NC	23	1	

Abbreviations: NA, not applicable; NC, no children.

^a^ Fisher's test.

^b^ Statistically significant.

CareHPV detected the presence of hr‐HPV DNA in 3.33% (16/480) of the samples that were analyzed. The bivariate analysis has demonstrated a significant association between the positive outcome of careHPV and the following risk factors: irregular menstruation (*p* = 0.005), alcoholic beverage consumption (*p* < 0.00001), tobacco chewing habit (*p* = 0.002), and the > 3 total number of children (*p* = 0.001). No significant association was observed between other epidemiological factors and the positive outcome of careHPV (Table 1). The age‐specific prevalence of hr‐HPV DNA was as follows: 20–30 years: 0.21%; 30–40 years: 1.04%; 40–50 years: 0.63%; 50–60 years: 1.25%; 60–70 years: 0.21%.

All variables associated with the hr‐HPV (careHPV) outcome were subjected to multivariable logistic regression. Women with tobacco smoking habit are at a 4.24 times higher risk of being hr‐HPV DNA positivity (OR‐4.24; *p*‐0.04; CI: 1.11–19.88). Women who consume alcoholic beverages have a 43.9‐fold increased risk of being hr‐HPV positive (OR‐43.9; *p* < 0.00001; CI: 10.83–407.68), whereas women who acquire urogenital tract infections have a 6.88‐fold increased risk of being hr‐HPV positive (OR‐6.88; *p* 0.008; CI: 1.76–33.62). Nevertheless, women with an irregular menstrual cycle have 11.26 times higher risk of getting HPV positive than with regular menstruation (OR 11.26; *p*‐ 0.004; CI: 2.21–66.56). Conversely, women who have more than three children are at a 10.7 times higher risk of being hr‐HPV positive (OR‐10.7; *p*‐0.003; CI: 2.41–63.21; Table 3).

Sixteen hr‐HPV DNA positive samples in the careHPV test were subsequently retested by the HC2 test for hr‐HPV DNA. All CareHPV positive samples yielded positive results in the HC2 test as well, with an RLU/CO value greater than or equal to 1. The recorded value for the positive CO RLU was 2275.

Cytological abnormalities were observed in the PAP smear of 37 out of 480 women, which accounts for 7.7% of the total. The specific details of the abnormal cytology were as follows: 2.5% (12/480) ASCUS, 3.50% (17/480) LSIL (CINI), 0.62% (3/480) HSIL (CIN II/III), 0.4% (2/480) AGUS, and 0.2% (1/480) AGC. The bivariate analysis has demonstrated a significant association between age (*p* = 0.026), tobacco chewing habit (*p* = 0.011), and aberrant cytology (PAP) outcome. Abnormal cytology did not exhibit any significant association with other epidemiological factors (Table 2). According to age, the prevalence of PAP positivity was as follows: 20–30 years: 0.83%; 30–40 years: 1.45%; 40–50 years: 2.71%; 50–60 years: 1.45%; 60–70 years: 1.25%.

**TABLE 2:** Sociodemographic, behavioral characteristics, and risk factor association with PAP outcome in women (bivariate analysis).

Characteristics	PAP negative	PAP positive	*p* ^a^
Age			
20–30	40	4	0.026^b^
30‐40	156	7	
40–50	152	13	
50–60	76	7	
60–70	19	6	
Tobacco habit			
No	290	16	0.011^b^
Yes	153	21	
Betel nut habit			
No	147	11	0.71
Yes	296	26	
Alcoholic beverages			
No	350	24	0.06
Yes	93	13	
Use of contraceptives			
No	302	26	0.85
Yes	141	11	
Urogenital tract infection			
No	293	25	1.00
Yes	150	12	
Urogenital tract infection of husband			
No	431	37	0.61
Yes	12	0	
Regularity of the menstrual cycle			
Irregular	48	6	0.28
Regular	395	31	
Use of sanitary pad			
Yes	277	20	0.37
No	166	17	
Age at first pregnancy			
10–20	140		0.91
20–30	237		
30–40	46		
40–50	1		
NA	0		
Total number of children			
< 3	274	23	0.91
≥ 3	148	12	
NC	21	2	
HPV status			
HPV negativity	434	30	0.00006^b^
HPV positivity	9	7	

Abbreviations: NA, not applicable; NC, no children.

^a^ Fisher's test.

^b^ Statistically significant.

**TABLE 3:** Multivariable logistic regression analysis of hr‐HPV.

Variables	Odd ratio (OR)	Confidence interval (CI)	*p*
Tobacco habit	4.24	(1.11–19.88)	0.044^a^
Alcohol habit	49.33	(10.83–407.68)	< 0.00001^a^
Menstrual cycle	11.26	(2.21–66.56)	0.004^a^
Urinogenital tract infection	6.88	(1.76–33.62)	0.008^a^
Use of sanitary pad	3.38	(0.78–16.61)	0.11
No. of children > 3	10.7	(2.41–63.21)	0.003^a^
Without Children (NO Pregnancy)	5.91	0.13–127.53	0.29

*Note:* #Total subjects = 480; #Positive cases = 16, #Negative cases = 464.

^a^ Statistically significant.

**TABLE 4:** Multivariable logistic regression analysis of PAP.

Variables	Odd ratio (OR)	Confidence interval (CI)	*p*
Age			
30–40	0.39	(0.11–1.60)	0.16
40–50	0.83	(0.25–3.21)	0.76
50–60	1.07	(0.27–4.69)	0.92
60–70	3.86	(0.88–18.64)	0.07
Tobacco habit	2.08	(1.03–4.26)	0.039^a^
Alcohol habit	2.12	(0.98–4.45)	0.049^a^
Use of sanitary pad	1.80	(0.82–3.97)	0.13

*Note:* #Total subjects = 480; #Positive cases = 37, #Negative cases = 443.

^a^ Statistically significant.

**FIGURE 1:**
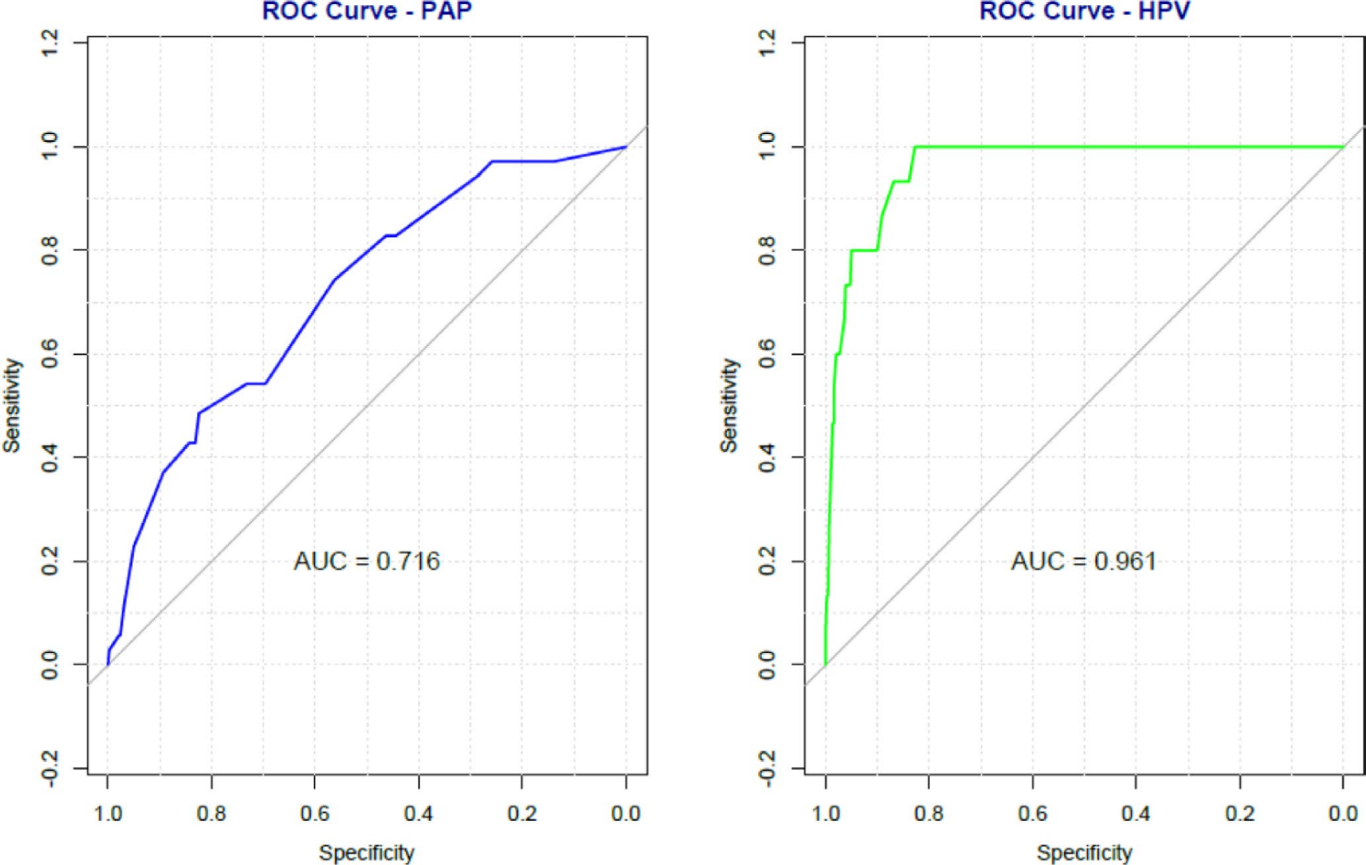
Receiver Operating Characteristic (ROC) curves for hr‐HPV and PAP.

We performed multivariable logistic regression for different variables with PAP outcomes. It has been shown that women with tobacco chewing habits are at a 2.08 times increased risk of being PAP positive (OR 2.08; *p*‐0.03; CI: 1.03–4.26). Women with an alcohol consumption habit have a 2.12 times higher risk for PAP positivity (OR 2.12; *p*‐0.04; CI: 0.98–4.45; Table 4). In our study, the prevalence of positive hr‐HPV DNA by careHPV and PAP is less than 10%; therefore, the odd ratio has been described as a risk ratio; 1.4% (7/480) of women had both positive PAP and positive hr‐HPV DNA outcomes. The cross‐tabulation between PAP and hr‐HPV outcome has demonstrated a highly significant association (*p* < 0.00006; Table 2). Receiver operating characteristic (ROC) curves were implemented to evaluate the internal validity of our logistic regression models. hr‐HPV had an area under the curve (AUC) of 0.9638 (CI: 0.9378–0.9898), while PAP had an AUC of 0.742 (CI: 0.6688–0.8152; Figure 1).

## 4 Discussion

The positivity of hr‐HPV DNA by careHPV among rural women in the Kamrup district was 3.3%. In previous studies conducted in Northern India, the prevalence of HPV‐positive outcomes was 2.9%, 6.4%, 3%, 8.6%, and 7.2% [16, 17, 18, 19, 20]. HC2 studies conducted in India have demonstrated hr‐HPV positivity in the following frequency: 12.7% and 17.1% in Northern India, 7.6%, 14.0%, and 12.6% in Southern India, 6% in Western India, and 16% in North‐East India [9, 10, 11, 12, 13, 14, 15]. The variations in the frequency of the careHPV positivity can be attributed to the chosen method for detecting HPV as well as the ethnic and cultural diversity within the studied population. The northern and southern regions of India conducted the majority of these studies. These studies indicate that there is still a significant lack of the careHPV and the HC2 tested hr‐HPV prevalence data among women in different regions of India. Accurate data on the prevalence of hr‐HPV obtained from clinically confirmed HPV DNA tests are essential for developing any national health strategy in India regarding the use of hr‐HPV DNA testing for the prevention of cervical cancer in women.

We observed a 7.7% prevalence for abnormal cytology (PAP) positivity; 1.4% of the women in our research exhibit both PAP and hr‐HPV positivity. We observed a strong correlation between positive hr‐HPV DNA status and positive PAP outcome in the same women (*p* = 0.00006). A study conducted in Delhi found a 7.9% prevalence of ASCUS positives. In our study, the prevalence of ASCUS positivity was 2.5% (12 out of 480 cases). In our study, we have found 43.75% sensitivity and 93.53% specificity of PAP test in detecting HPV. Another study conducted in India reported that the sensitivity of the PAP test for detecting CIN II+ and CIN III+ lesions was 62% and 41% respectively [21]. In contrast, cervical careHPV demonstrated a sensitivity of 85% for detecting CIN III+ lesions and 53% for detecting CIN II+ lesions [18]. 30% of women with positive hr‐HPV DNA had an 80% detection rate for CIN II+ lesions [20]. When it comes to PAP smear cytology, cytology‐based analyses are susceptible to inter‐individual observation variances that are connected to the length of experience that the technologist or pathologist has had with PAP.

Our findings suggest that individuals who chew tobacco are significantly more likely to be positive for hr‐HPV DNA (*p* = 0.002) and also positive for PAP (*p* = 0.011) compared to non‐users of tobacco chew. Our multivariable logistic regression has shown a significant association between the habit of tobacco chewing with both hr‐HPV DNA (*p* = 0.04) and PAP positivity (*p* = 0.039). The direct association between hr‐HPV infection and tobacco chewing and smoking has been established [22]. Nicotine dose and exposure length can influence immune cells' antigen signalling pathways and responses to bacterial or viral infections [23]. Enhanced risk of hr‐HPV infection associated with tobacco smoking may also have other residual confounding factors such as low risk HPV types or other sexually transmitted infections [24]. Compared to those who continue to smoke, the incidence of cervical cancer was reduced by approximately 50% among those who discontinued smoking for a decade [15].

In our multivariable logistic regression analysis, we observed a significant association of alcohol consumption with hr‐HPV DNA (43.9 times higher, *p* < 0.001) and PAP positivity (2.12 times higher, *p* = 0.04) among women.

Consuming alcohol raises the likelihood of cervical hr‐HPV persistence and CIN. In addition, individuals who consume alcohol have a greater likelihood of developing CIN 1 compared to those who do not drink alcohol [23]. In the North‐east India, indigenous ethnic groupings make their own alcoholic beverages using native ingredients. Most of these alcoholic beverages lack refinement, disregard proper hygiene standards, and contain a higher percentage of alcoholic ingredients, potentially harming women's health.

In our multivariable logistic regression analysis, we discovered that menstrual irregularity (*p* = 0.004) and urinary tract infection (*p* = 0.008) were identified as significant risk factors for hr‐HPV infection. The prevalence of HPV infection has been found to increase during the ovulation period [25]. These include estrogen's role in promoting cervical epithelial cell growth and maturation, which leads to the shedding of virus‐infected cells. Estrogens may also boost viral replication. Additionally, there is evidence of a midcycle reduction of mucosal immunity [24]. The persistence of HPV may have been facilitated by an increase in the levels of reproductive hormones, such as estrogen, during midstream menstruation, which resulted in an alteration in the immune and cytokine response to HPV [26].

We observed in the logistic regression model that having more than three childbirths is a significant risk factor for hr‐HPV DNA positivity (*p* = 0.003). Pregnancy‐induced cervical alterations involve an increase in the number of squamous metaplastic cells in the transformation zones. This makes women more vulnerable to hr‐HPV infection and increases their risk of developing CINs and invasive cervical cancer in the future [27]. It is suggested that more childbirths due to repeated hormonal changes and labour‐related trauma may increase the risk of hr‐HPV infection in women [28]. The projected incidence of HPV‐related cancers in India by 2025 may be approximately 1 21 000 cases [29]. A study of North‐East India Population‐Based Cancer Registries (PBCRs) has advocated investigating specific causes of cancer in the region and developing interdisciplinary research tools and approaches. This will facilitate the efficient implementation of cancer prevention and management strategies [30]. Efficient cooperation between the state government and different stakeholders, optimization of existing resources, and utilization of current healthcare technology may be the crucial factors for successfully implementing the HPV DNA testing strategy for cervical cancer prevention at the grassroots level in India [21].

Our study provided real‐world data on the hr‐HPV prevalence of the careHPV test among rural women in Assam. One of the limitations of our study was that it was limited to one district rural area, which may have restricted the representation of data regarding the prevalence status of hr‐HPV DNA among different ethnic groups in Assam. However, we can attribute this limitation to financial and logistical challenges. Additionally, the wider confidence intervals that were observed are a statistical indication of the inherent variability and randomness that are present in the estimation of parameters from a limited sample of the careHPV positive cases. This randomness is, in part, a result of the limited number of positive hr‐HPV cases, a common challenge in field studies and investigations. This emphasizes the need for additional research that uses sophisticated statistical modelling and larger cohorts to improve the generalizability and robustness of our findings.

Increasing knowledge about preventable risk factors, promptly recognizing signs and symptoms of CIN lesions and invasive cervical cancer, good genital hygiene practices, and conducting early screening and treatment for CIN II+ and CIN III+ lesions in both asymptomatic and symptomatic women can effectively reduce the occurrence invasive cervical cancer among women from urban areas of North‐East India. Implementing a self‐sampling technique for the careHPV test in rural parts of North‐East India could enhance the involvement and broader inclusion of women to effectively prevent cervical cancer. To successfully implement mass HPV vaccination for the prevention of cervical cancer in India, it is necessary to collect significant data on the hr‐HPV DNA status in women belonging to varied geographical areas and ethnicities.

## 5 Conclusion

We summarize from our study that alcoholic beverage use, chewing of tobacco, menstrual irregularity, urogenital tract infection and more than three children birth were found to be significant behavioural risk factors for an increased risk of hr‐HPV DNA positivity among rural women in the Kamrup district of Assam, North‐East India. We also found a significant association between an abnormal cytology (PAP)‐positive outcome with chewing tobacco and alcoholic beverage consumption. Further studies will be required to validate our current observation in a large cohort of women from different parts of Assam and North‐East India.

## Author Contributions

**Pallavi Sarma:** investigation, writing – original draft, data curation, formal analysis, methodology, writing – review and editing, visualization, validation. **Debabrata Barmon:** investigation, funding acquisition, writing – original draft, writing – review and editing, formal analysis, project administration, validation, visualization, data curation. **Avdhesh Kumar Rai:** conceptualization, investigation, funding acquisition, writing – original draft, writing – review and editing, visualization, validation, methodology, formal analysis, project administration, resources, supervision, data curation. **Amal Chandra Kataki:** project administration, writing – review and editing, resources. **Anupam Sarma:** investigation, writing – review and editing, visualization, methodology. **Upasana Baruah:** resources, writing – review and editing, methodology, investigation. **Lopamudra Kakoti:** investigation, writing – review and editing, visualization, validation, methodology. **Debanjana Barman:** investigation, writing – review and editing. **Ratnadeep Sharma:** software, formal analysis, data curation.

## Ethics Statement

The study was approved by the Institutional Ethics Committee of Dr. Bhubaneswar Borooah Cancer Institute (Ref No. BBCI /Misc‐119/MEC/177/2015). Dr. Bhubaneswar Borooah Cancer Institute Institutional Ethics Committee is registered in National Registry of Indian Council of Medical Research, Government of India (Registration No. ECR/1040/Ins/AS/2018).

## Conflicts of Interest

The authors declare no conflicts of interest.

## Data Availability

The authors have nothing to report.
